# Super-resolution microscopy reveals a golden kiss of death to mitochondria

**DOI:** 10.1038/cddiscovery.2016.38

**Published:** 2016-06-06

**Authors:** M Kodiha, D Maysinger, U Stochaj

**Affiliations:** 1 Department of Physiology, Montreal, Quebec H3G1Y6, Canada; 2 Department of Pharmacology and Therapeutics, McGill University, Montreal, Quebec H3G1Y6, Canada

Dear Editor,

Gold nanoparticles (GNPs) are established tools for research^1^ and promising agents for cancer therapy.^2^ Gold is inert and biocompatible; however, some GNPs also promote efficient tumor cell killing *in vitro*. GNPs are available in various sizes and morphologies; these are critical factors for the uptake and destruction of cancer cells (reviewed in Kodiha *et al*.^3^). In cellular models, GNPs can impair the function of different organelles and subcellular compartments. For example, GNPs often compromise the biological processes associated with nuclei or mitochondria.

To date, the mechanisms that trigger GNP-induced cell death are not fully understood, and the full spectrum of damage caused by different GNPs is often not known. This lack of knowledge is a major impediment to the therapeutic use of GNPs.^2^ Recent progress in cellular imaging provides a unique opportunity to generate this information and obtain new mechanistic insights into the action of GNPs. To this end, we focused on the analysis of MCF7 cells, an established model for breast cancer.

Breast cancer is the most common fatal cancer in women aged 20–59,^4^ and new therapeutic modalities are needed to improve treatment success. Among such novel therapies are GNP-based applications, which we tested on the breast cancer cell line MCF7. We have previously shown that small spherical GNPs (GN spheres, Supplementary Figure S1A) and gold nanoflowers (GN flowers) impinge on the nucleus and its compartments.^5,6^ The possible effect on other organelles was not examined.

Aside from nuclei, mitochondria are potential targets of GNPs;^3^ the nanoparticles may alter the mitochondrial organization, function or both. To address this question for GN spheres and GN flowers, we conducted super-resolution microscopy on controls and GNP-treated MCF7 cells (Supplementary Figure S1B). Stochastic optical reconstruction microscopy (STORM) uncovered – in the same cell – the changes of mitochondrial organization and function.

Elegant super-resolution studies have been performed for mitochondria. However, to our knowledge, a STORM approach has not been used to show the impact of GNPs on these organelles. Here, we applied this advanced imaging technique to define the GNP-induced mitochondrial damage. To this end, our experiments evaluated two different organelle parameters: (i) the organization and (ii) function of mitochondria. Tom20 served as a marker of mitochondrial organization. Tom20 resides in the outer mitochondrial membrane, where it is part of the protein import machinery.^7^ By contrast, MitoTracker CMX ROS (ThermoFisher Scientific, Mississauga, ON, Canada) informs on the electrochemical gradient across the inner mitochondrial membrane^8^ and thereby scores the cell’s ability to perform oxidative phosphorylation.

STORM revealed profound GNP-dependent changes in mitochondrial morphology and membrane potential (Supplementary Figure S1B). Interestingly, the mitochondrial damage induced by GN spheres differed from the changes caused by GN flowers. GN spheres diminished greatly the mitochondrial membrane potential (green). By contrast, GN flowers profoundly reduced the abundance and altered the distribution of Tom20 (red). Together, these results suggest that mitochondrial injury is determined by GNP properties (summarized in Supplementary Figure S1C).

## Conclusions

State-of-the-art super-resolution microscopy identified the specific damage GN spheres and GN flowers inflicted on mitochondria. To our knowledge, this is the first report that shows how morphological GNP differences can have distinct impact on mitochondrial organization and function. In Supplementary Figure S1C, we propose that the combined effects of GNPs on mitochondria and nuclei^5,6^ are critical for efficient cancer cell killing. The GNP-induced impairment of both organelles should be considered for the future improvement of GNP-based cancer therapies.
