# Supplementary material for: Super-resolution microscopy reveals a golden kiss of death to mitochondria
Source: Cell Death Discov. 2016 Jun 6;2:16038–. doi: 10.1038/cddiscovery.2016.38 (PMC4979438; doi:10.1038/cddiscovery.2016.38)
Supplement: Supplementary Information [file cddiscovery201638-s1.pdf]

Supplemental Information

The Supplemental Information provides a detailed description of experimental procedures and additional references for these methods that have not been cited in the main text.

Experimental Procedures

Gold nanoparticles. Small gold nanospheres and gold nanoflowers were synthesized and characterized as previously described [6]. Transmission electron microscopy (TEM) was used to visualize GNPs (Fig. S1A).

Cell culture and incubation with gold nanoparticles. MCF7 cells were grown in Dulbecco's Modified Eagle Medium (DMEM, Gibco), containing 12.7 mM glucose and supplemented with penicillin–streptomycin, 8% fetal bovine serum. Cells were grown on MatTek glass bottom culture dishes (3.5cm diameter; No. 1.0 cover glass). Treatment with small GN spheres or GN flowers was as described [6].

Incubation with MitoTracker Red and immunostaining. MCF7 cells were treated with MitoTracker® CMX ROS (M7512, Life technologies) as published [9]. In control experiments, the uncoupler FCCP (final concentration 2.5 µM; 10 min pre-incubation, present throughout the incubation with the MitoTracker dye) profoundly reduced MitoTracker® CMX ROS fluorescence (not shown). Accordingly, MitoTracker® fluorescence was appropriate to monitor changes to the mitochondrial membrane potential in our model system. To evaluate the impact of GNPs on MCF7 cells, samples were incubated with vehicle, GN spheres or GN flowers [5, 6]. MitoTracker® CMX ROS was then added directly to the medium (dilution 1: 5,000), and cells were kept for 30min at 37ºC. Tom20 was detected with primary antibodies (Santa Cruz, sc-17764; diluted 1:400) combined with Alexa Fluor® 647-conjugated secondary antibodies [9].

STORM. The use of superresolution microscopy in cell biology has been reviewed recently [10]. For the experiments presented here, images were acquired with an N-STORM microscope (Nikon), using a CFI APO TIRF 100X objective (1.49 NA) and 30,000 cycles. STORM data were processed to generate a 'blink' coordinate file to generate a one image reconstruction of all the blinks acquired for the 30,000 individual

images. Imaging was performed with buffer containing cysteamine, using a modified version of a published protocol [11]. In brief, the imaging buffer contained 39 mM Tris-HCl pH 8.0, 8.5 mM NaCl, 210 mM cysteamine, 1.16 µg/ml glucose oxidase, 70 µg/ml catalase and 7.7 % glucose.

Figure legend

Figure S1. Impact of gold nanoparticles on the organization and membrane potential of mitochondria.

A. GN spheres and GN flowers were used to alter mitochondrial properties and impair their function in MCF7 cells. The synthesis and physicochemical properties of GNPs have been described earlier [6].

B. Images were acquired for single cells by STORM (see Suppl. Information). Signals for Tom20 are shown in red; MitoTracker® CMX ROS is depicted in green. Scale bars are 10 µm. A 10 X magnified view reveals further details of the mitochondrial changes.

C. Simplified model for the effects of GN spheres and GN flowers on mitochondria. STORM showed that GN spheres caused a profound loss of membrane potential. On the other hand, GN flowers led to a severe change in mitochondrial organization. Our previous studies showed that GN spheres and GN flowers also damage the nucleus (N, blue).

Supplemental References

9. Kodiha M *et al. Nature Protocol Exchange* 2015; doi: 10.1038/protex.2015.009.

10. Fornasiero EF, Opazo F. *Bioessays* 2015; **37:** 436-451.

11. Dempsey *et al*. *Nat Methods* 2011; **8**: 1027-1036.
