# Supplementary figures and images for: Super-resolution microscopy reveals a golden kiss of death to mitochondria
Source: Cell Death Discov. 2016 Jun 6;2:16038–. doi: 10.1038/cddiscovery.2016.38 (PMC4979438; doi:10.1038/cddiscovery.2016.38)

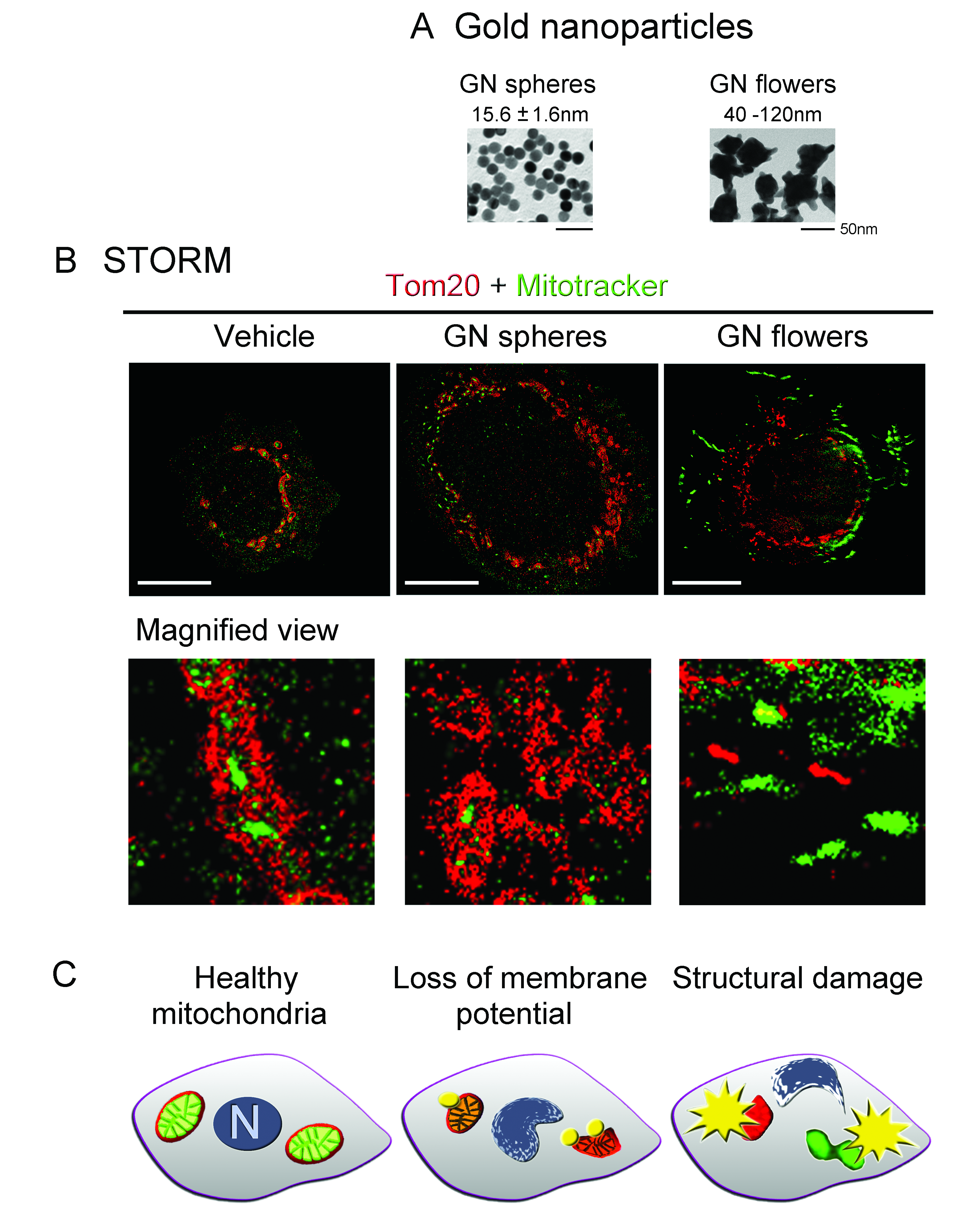

Supplement: Supplementary Figure S1 [file cddiscovery201638-s2.tiff]
